# Getting Greener with the Synthesis of Nanoparticles and Nanomaterials

**DOI:** 10.3390/nano12142452

**Published:** 2022-07-18

**Authors:** Robert Wojcieszak, Mohamed Nawfal Ghazzal

**Affiliations:** 1 University Lille, CNRS, Centrale Lille, University Artois, UMR 8181-UCCS-Unité de Catalyse et Chimie du Solide, F-59000 Lille, France; 2 Institut de Chimie Physique, Université Paris-Saclay, CNRS, UMR 8000, F-91405 Orsay, France; mohamed-nawfal.ghazzal@u-psud.fr

The nanoscale level is bridging the gap between molecular level and crystal-based solid-state structures. In this nanoscale range, classical laws of physics are no longer applicable by quantum mechanical rules [1], as in the case of bulk materials. Nanostructured materials may be defined as those whose structural elements, clusters, crystallites, or molecules have dimensions less than the 100 nm range [2,3]. However, generally, 1–20 nm particles are used for a wide range of applications in chemical transformations. The explosion in academic and industrial interest in these materials over the past decade arises from the remarkable variations in their electrical, optical, magnetic, and catalytic properties. Different methods have been used to synthesize metallic nanoparticles [4,5,6]. The most important are (i) chemical reduction, which involves the reduction of metal salts in solution or suspension by reducing agents; (ii) thermal decomposition by heating volatile metal compounds in an organic medium or gas phase, causing their degradation with the release of metals or their oxides in the dispersed phase; (iii) chemical vapor deposition in which the vaporized precursors are adsorbed onto a substance held at an elevated temperature; (iv) ultrasonic and microwave irradiations in which the direct interaction between microwaves and materials and this fact enables a uniform and fast heating of a sample; and (v) electrochemistry methods in which the particle size can be controlled by varying the electrical density, temperature, solvent polarity, and the distance between the electrodes. Unfortunately, harsh conditions or toxic solvents and precursors are often used. New greener methodologies are currently developed, which permit obtaining metal nanoparticles with narrow size distribution and controlled morphology. These methods are based on the Green Chemistry principles focused on the tendency to realize free-pollution synthesis or perform synthesis with only a few amounts of pollutant agents, using safe and renewable products and low energy demand.

Bimetallic Ag-Se nanoparticles can be prepared using extracts from *Ocimum tenuiflorum* [7]. The size of these nanoparticles can be controlled using different extract volumes. Moreover, Ag-Se nanoparticles displayed high stability, increased antioxidant activity, and biological compatibility at low concentrations. However, at higher concentrations, significant cytotoxicity and genotoxicity were observed. This study provided an important insight into the conditions required for the optimized synthesis of sustainable Ag-Se nanoparticles with interesting biological activity [7]. Due to their low toxicity and physical properties, gold nanoparticles are used for various applications. The synthesis of Au nanoparticles from plants is of great importance. In the work presented by Hassanisaadi et al. [8], 117 plant parts were screened originating from 109 different species. However, only 102 extracts demonstrated an Au^3+^ to Au^0^ bio-reduction. This study permitted revealing 37 new plant species [8]. In addition, the different sizes of Au nanoparticles can be obtained depending on the plant’s family. The study also included an interesting evaluation of the high potential of traditional East Asian medicinal plants for the synthesis of AuNPs. This is especially interesting from the environmental point of view for future applications in the cosmetic industry [8]. The synthesis of nanostructured materials from natural sources is highly desired, as prepared nanomaterials very often have unique chemical, physical, and biological properties.

Aziz et al. [9] showed that natural-based photoactive membranes can be easily prepared by the electrospinning method. This process is especially interesting because it enables the incorporation of natural minerals such as goethite in a polymeric nanofiber structure in one step, as prepared materials showed high biological activity in photocatalytic water remediation under visible-light illumination. The elaborated goethite composite nanofibers showed improved photoefficiency upon dye bleaching Escherichia coli and Clostridium perfringens deactivation. Moreover, due to the specific porous structure, these materials provide a promising application in continuous flow systems [9]. Small metallic nanoparticles such as Pt, Au, or Pd are also commonly used for catalytic applications. Several industrial processes are based on supported metals. One of these processes is glycerol aqueous phase reforming (APR) to produce hydrogen at low temperature [10]. Even if various noble metals were tested in this reaction, little attention was paid to Pt nanoparticles supported on TiO_2_. Fasolini et al. [10] developed a new method to obtain small and regular TiO_2_ nanospheres with high specific surface area. In their work, they optimized microemulsion methodology to obtain TiO_2_ with a peculiar high density of weak acidic sites. Moreover, once modified with Pt nanoparticles, these materials showed good activity in the glycerol reforming reaction. The authors evidenced the specific reaction pathways providing hydrogen yield at high glycerol conversion. This improved activity was correlated to the specific synergy between acid sites and Pt nanoparticles. In addition, this synergy was favored by the higher density of acid sites obtained thanks to the optimized microemulsion synthesis method and very small Pt nanoparticles with optimal surface dispersion [10]. The catalytic activity of metallic nanoparticles is very often governed by the method of preparation, which determines the particle size and structure of nanoparticles. This was demonstrated in the work of Scurti et al. [11]. The authors studied the effect of polymeric stabilizers on gold nanoparticle size and activity in hydrogenation of 4-nitrophenol. They showed that the gold colloidal solution is stabilized by the presence of a high amount of hydroxyl groups. However, a high quantity of the polymer at the metal surface inhibited the catalytic activity of gold due to the steric and electronic effects. In addition, it was demonstrated that the catalytic activity is dependent on the degree of polymer hydrolysis and a volcano plot was observed between the hydrolysis degree of the polymer and the apparent kinetic constant in the 4-nitrophenol hydrogenation [11]. The optimum catalytic activity was observed for the sample synthesized with polyvinyl alcohol hydrolyzed at 60%. These studies emphasized the crucial role of ligand–nanoparticle interaction in the catalytic mechanism of hydrogenation reaction [11]. The importance of the interaction between metal and the support in catalytic processes was also highlighted by Zhu et al. [12]. They studied a series of Zn supported on activated carbon catalysts. Prior to the catalysis, carbon materials were modified via thermal treatment in an ozone atmosphere to obtain different oxygen groups at the surface. A good correlation was observed between the catalytic activity and ratio of carboxyl and hydroxyl groups. They also demonstrated that the high catalytic activity of the prepared catalyst originated from the reduced strength of the increased capacity of acetylene adsorption. This work attracts attention toward revealing the crucial role of different oxygen-containing groups in the acetylene acetoxylation reaction, especially in the further industrial catalytic applications [12].
